# A mixed-methods study of pesticide exposures in Breastmilk and Community & Lactating Women’s perspectives from Haryana, India

**DOI:** 10.1186/s12889-020-09966-x

**Published:** 2020-12-07

**Authors:** Rukshan V. Mehta, M. A. Sreenivasa, Mathen Mathew, Amy Webb Girard, Sunita Taneja, Samriddhi Ranjan, Usha Ramakrishnan, Reynaldo Martorell, P. Barry Ryan, Melissa F. Young

**Affiliations:** 1 Doctoral Program in Nutrition and Health Sciences, Laney Graduate School, Emory University, Atlanta, GA USA; 2 Rollins School of Public Health, Emory University, Claudia Nance Rollins Building, 5th Floor, 1518 Clifton Road, Atlanta, GA 30329 USA; 3 Council for Scientific and Industrial Research – Central Food Technological Research Institute, Mysuru, India; 4 The Hubert Department of Global Health, Rollins School of Public Health, Emory University, Atlanta, GA USA; 5 Society for Applied Studies, Centre for Health Research and Development, New Delhi, India; 6 Gangarosa Department of Environmental Health, Rollins School of Public Health, Emory University, Atlanta, GA USA

**Keywords:** Organochlorines, Organophosphates, Pyrethroids, Pesticides, Breastmilk, In-depth interviews, Focus groups

## Abstract

**Background:**

Population growth which has resulted in a need for increased crop yields to sustain food security, in addition to the effects of climate change, have led to the widespread use of chemical pesticides. The indiscriminate use of pesticides has in turn led to contamination of the environment, food commodities and bioaccumulation in human tissues, particularly in agrarian regions of India including the northern state of Haryana.

**Methods:**

We conducted a pilot screening study to investigate the presence of organochlorine, organophosphate, and pyrethroid pesticides in breastmilk samples (*n* = 75) from Haryana, India. Pesticide analyses were conducted using gas chromatography mass spectrometry (GC-MS) for OC and OP pesticides and GC-electron capture detector for pyrethroids. The study was complemented by a qualitative evaluation of maternal and community perceptions, knowledge, attitudes and practices associated with pesticide use and risk of exposure (*n* = 30 in-depth interviews; *n* = 9 focus group discussions).

**Results:**

Analysis of breastmilk showed the presence of *p,p’*-dichlorodiphenyltrichloroethane (DDT) and *p,p’*-dichlorodiphenyldichloroethylene (DDE) in 4% (range: <LOQ - 28 μg/L) and 5% (range: < LOQ – 107 μg/L) of samples, respectively. No other pesticides were detected.

Our qualitative findings showed that community members commonly held perceptions of pesticides as medicines and poison but acknowledged their widespread use to ensure crop yields. Given the gendered engagement in farming in this setting, lactating women in study communities do not directly handle chemical pesticides, thus lowering risk of inhalation and dermal exposure.

**Conclusions:**

In our small sample, breastmilk pesticide concentrations were low and did not pose a risk to infants. Based on the persistent nature of many organic pollutants and reported widespread use, we recommend more comprehensive and longitudinal investigation of upstream pesticide contamination in the food supply and exposures among mothers and children.

**Trial registration:**

CTRI/2017/01/007636, Date Registered: 06/01/2017.

CTRI website: http://ctri.nic.in/Clinicaltrials/pdf_generate.php?trialid=17249&EncHid=&modid=&compid=%27,%2717249det%27

## Background

It is estimated that in India, annual losses in agricultural production due to pests, average US$42.66 million [1]. Chemical pesticides are used to protect against such pests and include organochlorines (OCs) such as dichlorodiphenyltrichloroethane (*p,p’*-DDT) and hydrochlorocyclohexane (HCH), organophosphates (OPs), including chlorpyrifos and pyrethroids such as cypermethrin [2]. Pesticides can be used as insecticides, herbicides (to kill weeds), fungicides (to control mold) and as rodenticides (to control rats, mice and moles) [3].

The demand for OP pesticides such as chlorpyrifos, profenofos and malathion and synthetic pyrethroids such as cyfluthrin, cypermethrin, deltamethrin has increased in India [4]. Pesticide use in the Indian state of Haryana is amongst the highest in the country, at approximately 0.62 kg per hectare [1]. Furthermore, a study conducted in Haryana showed that hardly 2–4% of farmers utilize masks, gloves, boots and other protective clothing during application of pesticides [5].

Organochlorine pesticides are also referred to as persistent organic pollutants (POPs). They bioaccumulate in the environment and become stored in adipose tissue due to their lipophilic, hydrophobic nature [6]. These pesticides exhibit low rates of chemical and biological degradation and are known to accumulate and bio-magnify in the food chain [7]. OPs and pyrethroids are readily oxidized or hydrolyzed by CYP enzymes or hydrolases in the body. The biological half-life for most OP pesticides is measured in hours to days and, unlike OCs, these do not bio-accumulate appreciably [8].

The Stockholm Convention on persistent organic pollutants (POPs) recommended elimination of hazardous OCPs such as aldrin, dieldrin and has restricted the use of DDT [9]. Hydrochlorocyclohexane (HCH) and its isomers were also recommended for elimination in 2009, due to emerging evidence of toxic effects on human health [10–13]. In India, although restrictions have been imposed on the use of OCs, since the 1990s, and their use has declined, DDT, and HCH continue to be used as household fumigants in vector control programs such as malaria [14, 15]. OCPs are also used in agriculture and for sanitation purposes [16, 17].

Adverse health effects of pesticide exposure include cancer and in extreme cases, death [18]. Additionally, these xenobiotic pollutants can readily be transferred by inhalation or dermal exposure and through the food chain, to human breastmilk [19–21]. Infants may be exposed to pollutants through breastfeeding and they may become stored in the body depending on the molecular affinity of the chemical and body composition of the exposed individual [22, 23]. Adverse effects of exposures to OC pesticides among children include cancer [24]. Exposure to OPs and pyrethroids have been linked to aberrations in cognitive, behavioral, sensory, and motor development in children [25–27]. Several studies have found pesticides in breastmilk samples from parts of India, although none have examined OCs, OPs and pyrethroid pesticides in human breastmilk from the agrarian north Indian state of Haryana [28–31].

The overall objective of this cross-sectional mixed-methods pilot study was to quantify levels of OC, OP, and pyrethroid pesticides in human milk samples from the Indian state of Haryana. Additionally, we aimed to examine community and lactating women’s perceptions of, knowledge, attitudes and practices (KAP) associated with farming and pesticide use.

## Methods

### Study population, mixed-methods data collection and analysis

**Fig. 1:**
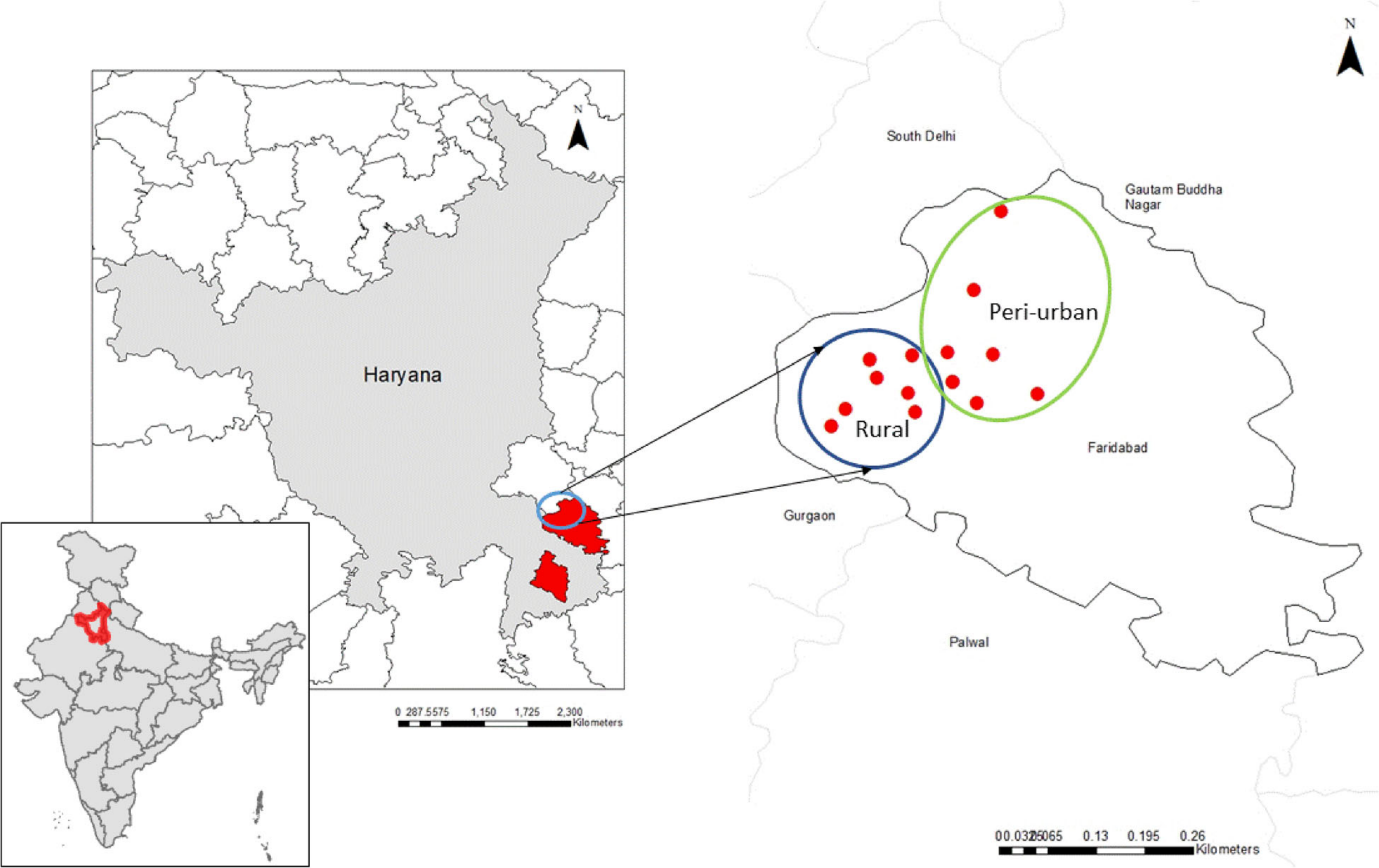
Location map of rural and peri-urban study sites in Faridabad district, Haryana, India The map was created using shapefiles available freely from datameet.org with ArcMaps software in ArcGIS 10.7.1.11595 (ESRI, 2019)

This mixed-methods study involved the use of quantitative and qualitative methods to understand breastmilk pesticide exposures among women in rural and peri-urban Haryana. This work was part of a larger study which looked at maternal malnutrition and its impact on lactation performance (*n* = 232). Mothers between 18 and 45 years, with children 2–4 months of age, who were currently breastfeeding were enrolled in the parent study. Participants were recruited from an existing pregnancy surveillance database in rural and peri-urban parts of Faridabad district, Haryana (Fig. 1). The study sites were chosen due to their proximity to New Delhi, the capital city of India, to allow for expedient transfer of breastmilk to the local laboratory for further  processing and to ensure integrity of the sample collection cold chain. Faridabad is a million-plus city, which experiences high rates of rural to urban migration, particularly from agrarian states such as Bihar and Uttar Pradesh. Estimated rates of in-migration average close to 55%, as people move in search of economic opportunities [32]. According to the National Family Health Survey – 4 (2015–2016), approximately 30% of children (< 5 years) in Faridabad district are stunted, 21% are underweight, 20% are wasted, and 14% of women (15–49 years) have a body mass index of < 18.5 kg/m^2^.

Parent study exclusion criteria included women who were not currently breastfeeding their children, those who were consuming tobacco products, and those who were not likely to stay in the region for a period of 2 weeks post-enrollment. The study protocol involved contact with the family over a 14-day period, with information collected on socioeconomic status, demographic details, maternal morbidity and well-being, physical activity, water scarcity, food insecurity, infant morbidity and a 24-h dietary recall. A wealth index was generated using principal component analysis (PCA) to determine tertiles of socioeconomic status and the Household Food Insecurity and Access Scale (HFIAS) was used to categorize households by food security status [33].

The study was approved by the Indian Council of Medical Research (ICMR), Society for Applied Studies Ethics Review Committee (ERC), and Emory University Institutional Review Board (IRB). Informed consent was read to all participants, who then provided written consent either using thumbprint for participants who were unable to sign or by written signature.

### Human milk sample collection

Breastmilk samples were collected on day 1 post-enrollment from study participants. Mothers were asked to manually express full milk from one breast, until empty. Breastmilk was collected and stored at 2–8 °C. Breastmilk energy content and lipid concentrations were estimated using a crematocrit machine (Separation Technology, Inc.). Crematocrit was expressed as a percentage of the length of total milk column, varied with lipid concentration of milk. Stored milk samples were kept at − 80 °C and shipped to external laboratories for additional analyses. A stratified random sample of 75 breastmilk samples, between 5 and 7 for each month spanning the 12-month collection period were analyzed for pesticides. A total of 26 organochlorines, 16 organophosphates and 11 pyrethroids were assessed in human milk samples.

### Chemicals

Magnesium sulfate (anhydrous), Primary Secondary Amine (PSA) and C-18 used for the extraction, were procured from Agilent Technologies (USA). Acetonitrile, acetic acid (glacial), and hexane (HPLC) were procured from Merck (India). Sodium acetate was procured from SRL (India). The PTFE syringe filters (0.45 um) used were purchased from Whatman (China).

#### Certified reference materials (CRM)

CRM for organochlorine pesticides comprised a mixture of 20 compounds viz. α-BHC, β-BHC, γ-BHC, Δ-BHC, heptachlor, Aldrin, heptachlor epoxide, *trans*-chlordane, *cis*-chlordane, endosulfan-I, endosulfan-II, endosulfan sulfate, 4,4′-DDE, 4,4′-DDD, 4,4′-DDT, dieldrin, endrin, endrin aldehyde, endrin ketone and methoxychlor; a herbicide mix of 5 compounds viz. atrazine, simazine, alachlor, metolachlor and butachlor and were procured from Sigma Aldrich (USA).

Individual standards for hexachlorobenzene, and several organophosphate pesticides including ethion, disulfoton, malaoxon, fenthion, phosalone, methyl parathion, methyl chlorpyrifos, ethyl parathion, fenitrothion, propazine and methyl paraoxon, were all procured from Sigma Aldrich (USA). Standards for malathion, acephate, phosphamidon, captafol, diazinon and monocrotophos were procured from Accustandard, Inc. (USA). A mixture of pyrethroid compounds including Cyfluthrin, L-cyhalothrin, cypermethrin-I, cypermethrin-II, cypermethrin-III, deltamethrin, pendimethalin, permethrin-I, permethrin-II, fenvalerate, and tefluthrin were also purchased from Accustandard, Inc. (USA).

### Breastmilk sample treatment

Pesticide extraction in milk samples was conducted using a modified and validated QuEChERS method, where 4 mL of milk was transferred to a 50 mL centrifuge tube to which a mixture of 3 g of magnesium sulfate, 0.5 g of sodium acetate and 10 mL of acidified acetonitrile (1% acetic acid in ACN) were added [8, 34]. The contents were mixed vigorously by shaking for 1 min using a vortex mixer (Spinix, India) and centrifuged (high speed centrifuge machine: Remi, R-24, India) for 5 min at 8000 RPM. Next, 8 mL of the clear supernatant was transferred to a 15 mL centrifuge tube containing a clean-up mixture of 1 g magnesium sulfate, 200 mg Primary Secondary Amine (PSA) and 300 mg of C-18. The contents were vortexed for 2 min, then centrifuged for 5 min at 8000 RPM, following which 5 mL of the resulting supernatant was transferred to a test tube and evaporated to dryness, using a flash (Turbovap LV from Calliper Lifesciences – USA) evaporator under a stream of nitrogen. The residue was reconstituted to 1 mL of hexane and filtered through a 0.45 μm filter into a gas chromatography vial. The samples were further analyzed by GC-MS and GC-ECD.

### Working standard solutions preparation

A stock standard mix of organochlorine pesticides and herbicides comprising a total of 26 compounds at 5000 μg/L concentration was prepared. Another stock standard mix comprising all organophosphate pesticides at 10,000 μg/L was prepared. The pyrethroid mixture was diluted to obtain a stock concentration of 20,000 μg/L. Working standards in the ranges of 10, 20, 40, 50, 75, 100 and 150 μg/L were prepared by following serial dilution techniques using hexane as the solvent and injected into GC-MS and GC-ECD to plot linear standard curves. Matrix matched standards were used for quantification by preparation in bulk from cow’s milk procured from different sources. Matrix matched blanks were checked to ensure lack of interference from any existing measured pesticides in our analysis. Matrix matched standards were prepared using blank matrix in the range of 10, 20, 30, 40, 50, 60 and 70 μg/L. These calibration curves were used for identification and quantification of pesticide residues in human milk samples.

### GC-MS and GC-ECD analysis

Pesticide residue analysis was performed using GC-MS (Model: 7890B) and GC-Electron Capture Detector (Model 6890) from Agilent Technologies, USA. Chromatographic separation was achieved using an HP-5 MS capillary column (0.25 μm, 30 m × 250 μm, Agilent Technologies India Pvt. Ltd). The samples were injected using a multi-mode inlet (MMI) in spitless mode, with injector set at an initial temperature of 180 °C, ramped to 270 °C at 40 °C/min; with an injection volume of 2 μL. The ion source temperature was set at 270 °C. Oven temperature was programmed as follows: initial temperature at 80 °C held for 5 min, with a run time of 5 min and ramped at a rate of 10 °C/min to 200 °C with a run time of 17 min. Ramp 2 was set at 5 °C/min and temperature of 290 °C, where it was held for 2 min at a run time of 37 min and ramp 3 was set at 10 °C/min and temperature of 300 °C, held for 6 min with a total overall run time of 44 min. Mass spectrometer conditions included electron impact ionization of 70 eV, a dwell time of 25 ms per ion transition, and a timed segment for Single Ion Monitoring (SIM) at 14th and 25th minute.

Pyrethroid pesticide residues were analyzed using optimized conditions in GC-ECD. Chromatographic separation was achieved using a HP-1 capillary column (0.25 μm, 30 m X 320 μm) from Agilent Technologies India Pvt. Ltd. Samples were injected in split mode, with an injection volume of 1 μL, at isothermal temperature of 260 °C for a total run time of 25 min. Pesticide concentrations are presented in μg/L and ng/g fat units. For ng/g fat calculations, breastmilk crematocrit measurements were used where available, and for one sample, crematocrit value was imputed by tabulating the median breastmilk fat value for the dataset.

### Quality control

All validation experiments were conducted using fresh cow’s milk samples procured from local and commercial vendors and served as controls. Samples were spiked with mixes of organochlorine (OC) pesticides, organophosphate (OP) pesticides at 10, 25 and 50 μg/L and pyrethroids at 50 μg/L to assess recoveries. Method specificity was also evaluated using fresh cow’s milk from different vendors. Chromatograms were checked for the presence of interfering matrix signals and corresponding corrections made (if any) during interpretation of results. Three quality control levels were used, lower QCs at 10 μg/L, middle QC at 25 μg/L and higher QC at 50 μg/L. Identification and confirmation of detected pesticides in samples was done by comparing the retention times of sample peaks with that of standards and abundancy of qualifier ions in samples using MSD (μg/L) calculated as: calculated concentration (μg/L)*dilution factor/sample volume. In samples where pesticides were detected using GC-MS, further confirmation was conducted by analyzing the sample using alternate column and detector (ECD), calculated as:

*standard concentration (*μg/L)*sample area*dilution factor/standard area*sample volume.

Limits of detection (LODs), described as the lowest concentration at which an analyte can be detected in a matrix with a signal to noise ratio ≥ 3.0, were found to be 10 μg/L for OC and OP pesticides; and 50 μg/L for pyrethroid pesticides. Limits of quantification (LOQs), describe concentration at which S/N ratio for quantifier ions ≥10 when comparing analytes from matrix matched calibration standards. For pesticides these ranged from 25 μg/L for OC and OP pesticides to 50 μg/L for pyrethroids.

Recoveries for all pesticides were within the acceptable range of 70–120%, with the exception of compounds including acephate, and monocrotophos which had relatively lower recoveries (25%) at lower QC levels (25 μg/L) [35]. All pesticide standard curve concentrations ranged between 10 and 150 μg/L with R^2^ values between 0.997–0.999, and matrix matched calibration curves had concentrations ranging from 10 to 70 μg/L with R^2^ values between 0.991–0.998.

### Qualitative data collection

To complement breastmilk pesticide analyses, a qualitative study was conducted between April and May 2018 to understand community perceptions of, knowledge, attitudes and practices associated with farming and risk of pesticide exposures. We used in-depth interviews (IDIs) and focus group discussions (FGDs) to elicit information on perceptions of food systems and environmental exposures. We collected information on a number of thematic areas, spanning the household food chain with a focus on homestead production, farming and use of pesticides and fertilizers. Study sites for the qualitative component (*n* = 10) were chosen purposefully and stratified to represent rural (*n* = 5) and peri-urban (n = 5) communities and to ensure representation of Hindu and Muslim households. In these communities, we conducted a total of 30 IDIs with lactating women who had children < 7 months of age and who were previously enrolled in the parent study. Of these, 14 IDIs were conducted with women from peri-urban communities, which in this region are located closer to Faridabad city. Most households in peri-urban communities do not have access to farmland nor do they practice farming. The remaining 16 IDIs were conducted with women from rural communities, all of whom owned or worked on farmland and came from livestock owning households. Rural communities sampled for this study are less densely populated and located further away from Faridabad city.

We also conducted a total of nine focus group discussions across three communities where IDIs were conducted. Two communities were rural, to ensure representation of religious and sociodemographic differences and one, peri-urban. In each community, we conducted one FGD with each of the following groups: mothers-in-law and elder women, elder men and fathers and frontline workers (FLWs). Frontline workers included Accredited Social Health Activists (ASHAs) and Aanganwadi Workers (AWWs), functionaries of the National Health Mission and Integrated Child Development Scheme, respectively.

Prior to commencing interviews, an interview guide was developed by the research team and included sections corresponding to household food production and associated chemical use (available in supplementary materials). Participants were interviewed by the first author in their households. The qualitative nature of this work, particularly within the cultural context of India, necessitated in many instances, that other members of the household be present. Where feasible, the interviewer requested that other members, particularly male family members excuse themselves to allow women to express themselves freely. Interviews were conducted until a saturation of themes was reached. We employed an emic approach, whereby the guide was revised and enhanced based on themes arising during the course of fieldwork. Each interview took approximately one hour, and participant fatigue and burden were key considerations throughout.

For community member focus group discussions, we enlisted the assistance of community elders in target communities to assemble mothers-in-law, fathers-in-law, fathers and FLWs. To avoid intrusion on work schedules, we spoke with FLWs beforehand and asked them to assemble at a day and time that did not conflict with their work responsibilities.

Detailed observations were recorded throughout the process, both in the context of household visits and community interactions. Several ethnographic tools were employed over the course of this work, including active listening [36], observations and note taking, formal and informal interviewing and critical self-reflection [37]. All IDIs and FGDs were audio recorded after acquiring consent and were transcribed verbatim from Hindi/Haryanvi to English. Transcriptions conducted by a trained Hindi-English translator were verified by the first author. All observations and reflections were recorded as field notes, which were also documented in Microsoft Word on a daily basis, during fieldwork.

A codebook was developed employing both *inductive* themes, which arose from respondents during interviews and *deductive* themes, which were identified based on the literature and while developing the interview guides [38]. Transcripts were open-coded manually using MAXQda (18.2.0, GmbH, 2018) and 10% were double-coded by an independent second coder to ensure inter-coder reliability. Thematic analysis was employed to analyze the data [39, 40]. We applied a knowledge, attitudes, practices (KAP) framework to our analysis of farming practices, food production habits and pesticide use among community members.

## Results

### Participant demographics and pesticide residues in breastmilk

Demographic characteristics of women for whom breastmilk pesticide residues were analyzed and their children are presented in Table 1. Average age (± SD) of women was 25.1 ± 3.75 years and median parity was 2.0. Maternal breastmilk fat content (38.8 ± 15.6 g/L) values are consistent with previous studies on human milk persistent organic pollutants (POPs) [29, 41, 42]. A total of 53 OCs, OPs and pyrethroids were analyzed in this study. We detected *p.p’-*DDT in three (4%) samples and *p,p’-*DDE in four (5%) samples. *p,p’*-DDT was detected in two samples at levels < limit of quantification (LOQ: 25 μg/L) and in one sample at 28 μg/L (679.6 ng/g lipid weight). In addition, *p,p’*-DDE was detected at levels of 34, 31, 107 μg/L, which converts to 1524.7, 1390.1, 2597.1 ng/g lipid weight and < LOQ in the fourth. The average BMI of women for whom pesticides were detected in breastmilk was 21.2 kg/m^2^ and mean % fat mass was 29.3.

**Table 1:** Household, maternal and child characteristics of *N* = 75 participants in Haryana, India

**Household Characteristics**	**N (%)**
**Religion**	
Hindu	51 (68)
Muslim	23 (31)
Other	1 (1)
**Caste**	
Scheduled caste	20 (27)
Other backward caste (OBC)	34 (45)
Other caste	21 (28)
**Paternal Occupation**	
Not working/retired/unemployed	4 (5)
Business/petty trader/self-employed	16 (21)
Salaried employee	38 (51)
Other	17 (23)
**Socio-economic Status**	
Low	25 (33)
Middle	25 (33)
High	25 (33)
**Residence**	
Peri-urban	58 (77)
Rural	17 (23)
**Food Insecurity**	
Food secure	62 (83)
Food insecure	13 (17)
**Maternal Characteristics**	**Mean (SD)/N (%)**
Maternal Age (years)	25.1 (3.75)
Maternal Education (years)	6.6 (5.3)
Parity	2.3 (1.3)
**Anthropometry**	
Weight, kg	60.0 (10.2)
Height, cm	152.3 (5.9)
BMI, kg/m^2^	21.9 (3.7)
**BMI categories, kg/m**^**2**^	
Underweight (< 18.5)	10 (13)
Normal (18.5–25)	52 (69)
Overweight (25–30)	11 (15)
Obese (> 30)	2 (3)
**Human milk fat content (g/L)**	38.8 (15.6)
**Current Dietary Patterns**	**N (%)**
Vegetarian	29 (39)
Non-vegetarian	40 (53)
Eggetarian	6 (8)
**Child Characteristics**	**N (%)**
**Child Sex**	
% Male	33 (44)
% Female	42 (56)
**Child Age (months)**	3.7 (0.60)
**Child Nutritional Status**	
% Stunted (length-for-age z-score < −2)	14 (19)
% Underweight (weight-for-age z-score < − 2)	12 (16)
% Wasted (weight-for-length z-score < − 2)	5 (7)
**Current breastfeeding practices**	
Currently exclusively breastfeeding	25 (33)

One sample in our study was found to have both DDT (28 μg/L, 679.6 ng/g lipid weight) and DDE (107 μg/L, 2597.1 ng/g lipid weight). This sample was in excess of maximum residue levels (MRL) for DDE set at 0.05 mg/kg by the FAO/WHO for commercial milk. *p,p’*-DDE is a marker of past exposure to DDT and its primary degradant [43, 44]. We calculated the ratio of DDT:DDE in the aforementioned sample to be ~ 1:4 or 0.26, indicating DDT exposure was recent (ratio < 5 indicates recent or ongoing exposure) [41]. This ratio decreases over time as DDT degrades [43, 44]. No other pesticides were detected in samples from our study. Three of the four women for whom *p,p’*-DDE was detected were from peri-urban communities, the fourth from a rural community. Three women were primiparous and one was multiparous (2 children). A broader comparison of findings from studies conducted on pesticide residues in Indian breastmilk samples between 2006 and 2018 are presented in Table 2.

**Table 2:** Comparison of studies examining pesticides in breastmilk from India (2006–2018)

Reference, location of study	Samples analyzed (n)	Sample type & [range] detected	LODs, LOQs, ADIs	Extraction protocol, matrix volume & analytical method
Kumar, Dayal, Shukla, Singh, Joseph, 2006 [45] Agra, India	32	*Breastmilk:* DDTs: 0.17–0.179 mg/kg HCH: 0.123–0.131 mg/kg	*LODs:* 0.001 μg/mL *LOQs:* 0.01 μg/mL	USEPA method (1980) [46] and Ejobi et al., 1996 [47] 20 mL milk GC-ECD
Kumar, Baroth, Soni, Bhatnagar & John, 2006 [17] Rajasthan, India	50	*Breastmilk:* Rural: 5.0625 mg/L Urban: 3.243 mg/L *Blood:* Rural: 4.608 mg/L Urban: 3.777 mg/L	Not reported	Takei et al., 1983 (milk) [48] Bush et al., 1984 (blood) [49] GC-ECD
Devanathan et al., 2009 [50] New Delhi, Mumbai, Kolkata, Chennai	64	*Breastmilk:* DDT: 47–1200 ng/g lw HCH: 6.3–1900 ng/g lw	Not reported	Kuniseu et al., (2004) [51] 10 g milk GC-ECD
Mishra & Sharma, 2011 [52] Assam, India	205	*Breastmilk:* ΣDDT: 2870 ± 1570, 3210 ± 2080 ng/g lw ΣHCH: 2330 ± 1160, 2720 ± 2140 ng/g lw	*LOD:* 0.001 ng/g *LOQ:* *DDT:* 0.01–0.2 ng/g lw *HCH:* 0.02–0.3 ng/g lw *ADIs:* DDT: 16–18 μg/kg bw/day HCHs: 13–15 μg/kg bw/day	Dua et al., (1997) [53] GC-ECD
Maurya, Kumar Joseph, 2013 [54] Uttar Pradesh, India	10	*Breastmilk:* ΣDDT: 158 ng/mL	*LOD:* 0.001 μg/mL *LOQ:*0.01 μg/mL	Ejobi et al., 1996 [47] 5 mL LC-ECD
Bedi, Gill, Aulakh, Kaur, Sharma & Pooni, 2013 [29] Punjab, India	53	*Breastmilk:* ΣDDT: 1914.2 ng/g lw (63% DDE) ΣHCH: 199.6 ng/g lw	*LOD:* 1 ng/g on whole milk basis	Battu et al., (2004) [55] 5 mL GC-ECD Confirmed with GC-MS
Sharma, Gill, Bedi & Pooni, 2014 [30] Punjab, India	127	*Breastmilk:* DDE: ND – 43.19 ng/g (9 > PTDI) HCH: ND – 133.88 ng/g (11 > PTDI)	*LOD:* 1 ng/g on whole milk	Battu et al., (2004) [55] 5 g milk GC-ECD Confirmed with GC-MS
Sharma, Sharma, Chandel, & Wise, 2016 [56] Himachal Pradesh	153	*Breastmilk:* DDE: BDL – 0.064 mg/L DDT: BDL – 0.018 mg/L Mean ΣDDT: 0.145 mg/kg fat (*n* = 27% +) Mean DDE: 0.138 mg/kg fat (n = 27% +) Mean DDT: 0.005 mg/kg fat (*n* = 1.31% +)	*LOQ:* 0.005–0.001 mg/kg	QuEChERS method developed 10 mL milk GC-ECD Confirmed with GC-MS (SRM)
Bawa et al., 2018 [31] Bathinda, Ludiana, Punjab	150	*Breastmilk:* DDT: 415.3–519.2 ng/g lw ΣDDTs: 519.2 ± 1017.4 ng/g (ND – 4583.1) 415.3 ± 846.32 ng/g (ND – 3212.5)	*LOD:* 8–20 ng/g	Battu et al., (2004) [55] 10 mL milk GC-ECD Confirmed with GC-MS

### Qualitative study of household food production: knowledge, attitudes & practices related to pesticides and fertilizers

#### Demographic characteristics

Demographic details of participants of in-depth interviews and focus group discussions are presented in Table 3. Approximately 30% of women in our sample practiced farming in their current residence and 43% had historically practiced farming in their pre-marital households. Families in our study communities owned small plots of land, averaging approximately 2 acres. Main crops farmed in these communities include wheat, sorghum, pearl millet, animal feed for buffalo, and mustard seeds. A few households noted growing rice and maize. Most households practiced subsistence farming, with a few producing enough wheat for commercial sale. Primary irrigation facilities for farming come from groundwater borewells and pipes. Seeds for cultivation are primarily procured from the market, however some families described using seeds planted in previous seasons, after traditional preparation. Soil is cultivated using tractors and other mechanized equipment.

**Table 3:** Demographic characteristics of in-depth interviews and focus group discussion participants

Population	Peri-urban	Rural
**In-depth Interviews (n = 30)**		
**Lactating Mothers (#)**	14	16
Maternal age (mean)	26.3	23.9
Religion (n)	14 – Hindu	8 – Hindu, 8 - Muslim
**Focus Group Discussions (n = 9)**		
**Mothers in-law/Women (#)**	6	16
Age (mean)	45.7	59.2
Religion (n)	6 – Hindu	10 – Hindu, 6 – Muslim
**Fathers in-law/Fathers (#)**	7	17
Age (mean)	52.4	47.5
Religion (n)	7 – Hindu	8 – Hindu, 9 - Muslim
**Frontline Workers (#)**	5	12
ASHAs	–	6
AWWs	5	6
Age (mean)	36	32.9
Religion (n)	5 - Hindu	11 – Hindu, 1 - Muslim

#### Qualitative analysis

Our qualitative research question examined *perceptions* of, *knowledge, attitudes* and *practices* associated with farming, pesticide handling and use among community members and lactating women in Faridabad, Haryana. The results of the analysis and interpretation of our qualitative data are presented as key themes, listed below:

- Perceptions and attitudes towards pesticides as medicine vs. poison and perceived health effects of pesticides and fertilizers
- Knowledge and practices related to pesticide handling and use
- Gendered engagement in use of pesticides and fertilizers

### Perceptions and attitudes towards pesticides as medicine vs. poison and perceived health effects of pesticides and fertilizers

During focus group discussions, several community members across rural and peri-urban sites described pesticides as “*medicines for crops*” as well as “*poison*”. Similar perspectives were held for fertilizers. These dichotomous views of pesticides and fertilizers informed an understanding of the benefits and harms of such chemicals and community members’ perceptions and attitudes towards them. They noted that such chemicals are essential to ensure adequate yield of crops and hence necessary to avoid hunger. Yet, people were torn between the benefits of pesticides for adequate crop yield and their potential and perceived deleterious effects on humans.

*“To protect the crops from insects otherwise the insects will infect the roots and the crops will die. Nothing happens it only kills the insects in the wheat and once we eat, we wash it so there is no effect.”*

- FGD with mothers-in-law (peri-urban)

*“Four years later, when more money came, we started using Sipra, which has made the crops poisonous. Because this medicine is poisonous, if we eat it, it can cause problems.”*

- FGD with mothers-in-law (rural)

Despite this recognition, there was an overall sense of futility at a lack of alternatives or options.

*“These medicines are all poison, even then we are compelled to use.”*

- FGD with mothers-in-law (rural)

With regards to perceived health effects, most community members were wary of pesticide and fertilizer use and their implications on human health. Some community members noted that children’s height remains “short” because of exposure to chemicals. Community members noted that chemicals can be transferred from maternal diet and breastmilk to children, which can in turn adversely affect their health and well-being.

*“It affects the lactating mother, we put potash for growth and mother feeds milk to their child to strengthen bones of the child. But if the mother will eat that food then it will go to the child as well. We put poison in the crops then it will affect the body, it weakens the body.”*

- FGD with fathers-in-law (rural)

*“One can become unconscious and suffer from headache. Diseases can occur, children can be aborted. I put the medicines so that we can eat. Because of these tablets and medicines, diseases are occurring. Some are getting appendicitis, hernia. What to do in that, nothing can be done, we give rice and milk to eat. If the boy is small and girl is tall then they will not get good match for getting married.”*

- FGD with fathers-in-law (peri-urban)

*“There is no option. To fill the stomach and for the growth of cereals, urea needs to be added. It is affecting the growth of the children. Whatever mother and father are eating, children are also eating that only. Now 5-year-old children are not growing properly. Urea and manure are not proper now a days.”*

- FGD with fathers-in-law (rural)

Community members also described perceptions of the effects of pesticide and fertilizer use on the overall health of the population, and their role in chronic diseases such as cancer and obesity.

*“Maximum occurrence is there for cancer, TB, everything is happening because of these chemicals, weak eyesight, obesity”*

- FGD with mothers-in-law (rural)

*“Buffalo eats fodder and the fodder contains urea and milk will be formed. From that only urea is put in complete farm and all the eatables contain urea. When government is selling such things then they should sell the products that are beneficial, in place of urea they can give us something else. Acidity, blood pressure, heart attack as it has direct effect on the heart, if more urea is put then it has worse effect. They are putting more urea as knowledge is not there, proper training is not there.”*

- FGD with fathers-in-law (peri-urban)

As with pesticides, there remains an understanding of the possible ill-effects of excess fertilizer use and consumption, and a simultaneous sense of dependence on these chemicals to ensure crop health and adequate yields. Elder community members were able to describe a transition to use of modern-day synthetic chemicals as replacements for natural manure. They cited that the natural products (i.e. cow dung) are healthier for humans but not effective at producing adequate crop yields.

### Knowledge and practices related to pesticide handling and use

Both elder and lactating women during FGDs and IDIs, described the use of “*keetanashak dawa*” or insecticides and “*kharpatwar*” in order to kill weeds. When probed regarding their knowledge including names of specific pesticides used, women were able to identify DDT and Sipra, a powder which is sprayed on crops. Some elder women were aware of regulations around procurement of chemicals.

*“We do not get medicines just like that, before they give us, they verify how much land is there, that much medicine only they will issue. If you ask for 10 acres of land and you have only 2 acres, then you won’t get medicine for 10 acres.*

- FGD with mothers-in-law (rural)

Pesticide spraying is common practice in study communities, however limited if any personal protective equipment (PPE) such as masks and gloves are used and can include a cloth which is tied over the mouth, so the spray isn’t inhaled. Additionally, slippers or shoes are worn on feet. When questioned about the purpose and intention behind the use of pesticides, communities described issues with insect infestation, namely “*Siroli* or *termites*”, and noted that “medicines” are used to protect crops from pests.

*“Fungal infection and insects do not harm the crops and they grow healthy;it protects the crops from being destroyed.”*

– HH IDI with lactating woman (rural)

They also noted the use of “*dung and manure*” to protect crops from insect infestation and related damage, specifically identifying dialkylphosphates (DAP), zinc and urea. Like pesticides, fertilizers are generally applied to fields, manually, with bare hands or using gloves, with limited PPE. Most women had little knowledge of the exact location from where pesticides are procured and noted that they are stored away from the household, on the farm, where children and other household members cannot access them.

Additionally, women noted that the chemicals are disposed of, away from the household. Several community members and women were aware of incidences where these chemicals had been used for human consumption, leading to death. This created a general reticence around their handling, storage and use, with the task being handled primarily by elder males within the household.

*“After spraying the medicine, it is thrown in the jungle along with its tumbler. Whenever it has to be sprayed, it is brought out, otherwise it is not brought out. If a fight happens, in anger somebody may eat it and die. No one keeps it in their house, farm doctor gives this medicine and it is stored on the farm.”*

– HH IDI with lactating woman (rural)

### Gendered engagement in farming and use of pesticides and chemicals

Cultural norms, particularly in some Hindu communities within this region dictate that younger women do not leave the home. Although lactating women in several communities do go out to farm, when other community members were asked, they noted that pregnant women do not engage in farming as they risk “aborting” their child. Additionally, community members noted that many women do not farm due to personal safety concerns.

*“Like this also daughters and daughter in laws do not go to the farm, there is no safety”*

- FGD with mothers-in-law (rural)

Women in rural Muslim households do engage in farming activities, which primarily include watering the soil and harvesting, described as “*crop cutting*”. This is likely due to disparities in socioeconomic status, where households within these communities may not be able to afford outside labor, thus necessitating women’s engagement. Although collectively, farming remains the domain of male members of the household in study communities.

*“No, in our house ladies are not aware about farming. Gents are aware about farming”*

- HH IDI with lactating women (peri-urban)

It was noted that lactating women are not directly involved in the handling and use of pesticides.

*“No, it does not affect us as we do not spray the medicine, it is sprayed by some other person”*

– HH IDI with lactating woman (rural)

Rather, most women noted that their families hire outside labor to spray pesticides using machines, though their husbands or fathers-in-law also engaged in pesticide spraying. As a result of their limited engagement in farming and pesticide handling, lactating women’s knowledge of pesticides was rudimentary.

## Discussion

This pilot study was undertaken to detect pesticides in a cross-sectional sample of human breastmilk, and to understand knowledge, attitudes and practices of lactating women and communities with regards to pesticide and fertilizer use. Pesticide exposure in breastmilk in our study was low, and only 4 samples showed any detectable traces of OCs.

Prior studies conducted in regions in close proximity to our study site, including New Delhi, the national capital of India have found organochlorine pesticide concentrations in human breastmilk samples at levels between 15.8 ± 8.48 μg/L for total-Hexachlorocyclohexane (HCH), 1.8 ± 1.12 μg/L for DDT and 2.9 ± 2.54 μg/L for DDE [28]. Bedi et al., [29] reported mean ƩDDT (DDT + DDE) concentrations in breastmilk of 1.914 μg/L lipid weight, collected from the state of Punjab.

Another study conducted in the north Indian state of Punjab found mean breastmilk concentrations of *p,p’-*DDE at 0.00056 ± 0.0042 μg/L [30]. More recently, Bawa et al., [31] found *p,p’-*DDE at 0.407 ± 0.885 and 0.345 ± 0.815 μg/L lipid weight from the north Indian cities of Bathinda and Ludhiana. They also detected ƩDDT at 0.519 ± 1.017 and 0.415 ± 0.846 μg/L lipid weight in both cities respectively [31].

Mean values for the samples where DDT (17.7 ± 8.9 μg/L, *N* = 3) and DDE (46.1 ± 41.7 μg/L, *N* = 4) were detected in this study are in line with other studies conducted in India. For samples where pesticides were detected, it is likely that women had historical exposure to OCPs. In addition, higher maternal %fat mass and increased accumulation of OCs in adipose tissue, alongside variances in dietary patterns may also explain low levels of OCs observed in these select samples. For the sample where both *p,p’*-DDT and *p,p’*-DDE were detected, recent exposure may be explained by dietary habits or indoor residue spraying conducted in the participant’s peri-urban dwelling. However, given such a low sample with exposure, we were unable to fully examine key determinants further.

The limit of detection (LOD) in our screening study is also higher (10 μg/L; LOQ of 25 μg/L for OCs) than those in the published literature from India, which range between 0.008–0.02 μg/L [31]; 0.001 μg/L (OCPs) and 0.002 μg/L (OPs and SPs) [30]. It is important to note that our LODs (10 μg/L) were below maximum residue levels of 0.05 mg/kg (50 μg/L) set by the FAO/WHO for DDTs in milk, thus allowing us to detect trace concentrations and values of public health significance [43]. Based on our findings, it is possible that trace levels of other pesticides were not detected but are present in breastmilk. In addition, it is also likely that the gradual phasing out of these compounds in India has resulted in lower concentrations of residues in breastmilk.

Studies of pesticides in breastmilk from other countries, conducted in the last decade, have reported DDTs at concentrations ranging between 0.119 μg/L fat in Japan [57], ƩDDT at 0.063 μg/L fat for samples collected from Australia [42]. DDTs in breastmilk from Poland were reported at 1.621 μg/L fat [58], 14.46 μg/L fat in Ethiopia [22] and 1.365 μg/L in the USA [8]. In a systematic review of the international literature conducted between 1980 and 2013, Pirsaheb et al., [59] found breastmilk pesticide concentrations of OCs to range between 19 and 3210 ng/g for DDT, in India and Taiwan, respectively. In Africa, these authors note that DDT was detected in breastmilk at average concentration of 1163 ng/g from Tunis [59].

Approximately 90% of exposure to pesticides occurs via dietary intake as opposed to dermal or inhalation routes [60]. Several studies conducted in northern India have found residues of DDT, DDE, and other organochlorine pesticides in various staple crops such as rice (median value of 0.01 mg/kg; 90% of samples > MRL of 0.1 mg/kg) and produce including cauliflower, tomatoes,  radish, brinjal, spinach among others [61–63]. Testing food samples in addition to breastmilk is recommended for future studies.

Based on our qualitative findings, lactating women in study communities do not engage directly in pesticide spraying and use. That does not preclude secondary inhalation, ingestion or dermal routes of exposure. For women who do engage in farming, training on integrated pest management practices and additional agricultural extension services may be warranted [64].

Concerns around the consumption of pesticides to induce suicide, described by women and community members in our study have been reported elsewhere in India [65]. Several participants described “means restriction” or limiting access to pesticides to avoid potential self-harm, as an important driver for storage of chemicals away from the household [66]. This in turn, safe-guards lactating women and children from direct exposure to harmful pesticides. Dichotomous perceptions of pesticides as “medicines” and “poison” among community members, do not prevent their use. There is a recognition that such chemicals are necessary to ensure adequate crop yields, despite potential harm. For peri-urban samples in the study, it is possible that women have had historical exposure to POPs in their pre-marital households, where farming is practiced. The study of long-term and intergenerational risk of exposure using longitudinal study designs, with larger sample sizes is therefore warranted.

In addition to pesticides, further research is needed to understand the implications of extensive fertilizer use in study communities, with concomitant surveys of biomarkers of exposure in human samples. Community members equated exposure to chemical fertilizers such as urea and potash with several deleterious health effects and stunting in children. More research is needed to validate these concerns while accounting for the essential nature of such chemicals in maintenance of crop yields and to ensure food security.

## Conclusions

Our study suggests a need for further investigation of food systems and environmental exposures including pesticides and fertilizers, in peri-urban and rural communities of Haryana, India. Although pesticides do not appear to be a major concern in breastmilk in this small sample, it is prudent to investigate their presence upstream in the food supply chain. As women do not engage directly in farming, their risk of direct occupational exposure remains low.

Exclusive breastfeeding is recommended for children < 6 months of age, and breastmilk remains an essential source of nutrients and immune factors for growing babies. Based on our findings, risk of pesticide exposure for children via breastmilk is not a major concern in this population, however testing of pesticide residues in animal milk, dairy products, complementary and weaning foods is recommended based on results from other studies in the region [4, 67].

## Data Availability

The datasets used and/or analyzed during the current study are available from the corresponding author on reasonable request.

## Abbreviations

DDE: Dichlorodiphenyldichloroethylene
DDT: Dichlorodiphenyltrichloroethane
LOD: Limit of Detection
LOQ: Limit of Quantification
MRL: Maximum Residue Limits
OCs: Organochlorine Pesticides
OPs: Organophosphate Pesticides
POPs: Persistent Organic Pollutants
